# Harris Dental Association

**Published:** 1869-05

**Authors:** 


### HARRIS DENTAL ASSOCIATION.

The Second Annual Meeting of the Harris Dental Association was held at New Holland, Lancaster County, Pa., on Thursday, May 6. The attendance of so large a number of the members was sufficient evidence of their unabated interest in the prosperity of the Association, while the reports of the Secretary and Treasurer disclosed a favorable condition of its affairs. The election for officers resulted as follows:

President—Dr. S. Welchens.

Vice President—Dr. P. W. Heistand.

Secretary—Dr. Wm. Nichols Amer.

Treasurer—Dr. J. G. Moore.

Executive Committee—Drs. McCalla, Webb and Hoffer.

Delegates to State Dental Society—Drs. Heistand, Moore and Webb.

Delegates to the American Dental Association—Drs. Amer, Hoffer and McCalla.

The retiring President, Dr. John McCalla, delivered the annual address and presented certificates of membership to those present. After the transaction of the ordinary business and the reading of an essay by Dr. Welchens, the Association adjourned to meet in August next, at Euphrata Mountain Springs. Respectfully yours,

JOHN McCALLA.

NEW YORK, May 7th, 1869.

Dr. J. TAFT—*Dear Sir:* In accordance with your kind proposition made at Niagara, I send you for publication the Constitution, Order of Business, and Rules of Order of the American Dental Association, as amended by their Committee.

The amendments to the Constitution were proposed last year, therefore we shall have either to reject or adopt them at our approaching session, any material alteration being contrary to our existing Constitution.

The Order of Business was adopted last year, and will be our law for this year, unless altered by a three fourths vote of all the members present.

The Rules of Order are substantially those adopted by the New York State Dental Society, after pretty thorough consideration.

It is probably best to publish these proposed amendments before the meeting; as, if they are unworthy, they will be then fully known and can be rejected; if they are worthy, they can be adopted without long consideration, and the few who may prefer to have such laws as shall give to certain individuals, more than their due share of influence in the Association, will not be able to materially alter the views which will already have been formed.

Yours very respectfully,

E. A. BOGUE, *Chairman of Committee.*

